# The Physical Condition of the Teeth. The Causes Which Determine It, and as a Consequence, the Differences in the Liability of These Organs to Decay

**Published:** 1853-10

**Authors:** A. A. Blandy


### ARTICLE VIII.

*The Physical Condition of the Teeth. The causes which determine it, and as a consequence, the differences in the liability of these organs to decay.* By A. A. Blandy, M.D., D.D.S.

The causes which determine the physical condition of the teeth, and as a consequence their liability to disease, constitute a subject of study in which every dental practitioner should feel interested. The public have a right to expect from him such advice as would, if followed, not only contribute to their health and preservation, but also, to their restoration, when they become the seat of actual disease.

It is most lamentable to witness the almost total want of correct doctrine and knowledge, pertaining to the true cause of disease, the relative condition, coequal formation, growth, connection and nourishment of the teeth, during the period in which they, as well as the whole body, are being formed and brought into practical life. It is doubtless, the fact, that ignorance concerning these points, permits the existence of a most fearful and growing cause of disease in the general system, as well as a *consequent* and *steady* increase in the diseasès of the teeth, even more, perhaps, than results from bad and unskillful operations with the imperfect condition of the operative department of our art, on the one hand, or the improper administration of medicine arising from the imperfections of that science, and its frightful accumulations of petty quackeries on the other.

Were the laws properly understood which regulate the health of the body, the necessity for remedial applications would be measurably wanting, and vigorous health would greatly predominate. When society was less artificial, man's wants were few and easily supplied. Nature was not taxed to dispose of not only unnecessary but poisonous indulgences in food. His occupation produced the needed exercise, as well as a strong and healthy appetite, requiring no other stimulant than wholesome food, to sustain the system. The functions of the body performed their duties regularly, a robust constitution was the effect of such temperance, with an almost total exemption from the epidemical and local affections, and the many minor evils that mankind are now laboring under.

The comparison of the present condition of mankind, is obviously bad and discouraging. From birth up to manhood or adult age, we are tottering on the brink of the grave, and time makes a sad impression upon the man, instead of ripening to a full development his physical nature.

Instead of the endurance and prowess of the ancient Athenian and Roman, we are dwindling down towards Swift's inimitable conception of small men.

The deterioration is not applicable to a part or peculiarity only, but is general and evidenced in each and every organ which constitutes a part of the body, not equally evinced but relatively affected, depending upon the temperament, hereditary proclivity and local influences surrounding the subject. The teeth, when considered in their inseparable relationship and minute, indicative structure, most forcibly establishes this position, to the consideration of which we shall confine our remarks.

That diseases of the teeth were rare occurrences with the ancients, is beyond all doubt, in view of the facts found either in history or in regular treatises of anatomy, medicine or surgery. We might cite, as an example, the leaden instrument mentioned by Erasistratus, which was found suspended in the temple of Apollo. Everything leads to the belief, that the temporary teeth furnished the most frequent cases for extraction. Hippocrates' attention was more directed towards the eruption of teeth; he merely confessed, that they were bony organs, a little more subject to vicissitudes from exposure, and that certain conditions of the teeth were symptomatic of certain maladies. Aristotle, the best of ancient anatomists, and the first to devote any close investigation to the teeth, confines his attention to their comparative anatomy, without a single expression which would indicate that they were ever in a diseased condition.

Celsus gives the first clue to any painful affection or morbid condition of the teeth, by reporting the advice given by Archigeneus, to perforate the tooth with a small trepan when it is the subject of pain. Then comes Pliny, who tells the fabulous story of the Cæsarian soldiers, who lost their teeth by drinking from a certain fountain.

Galen, Paracelsus, Acteus and others, describe their anatomical relationship, but mention little or nothing of their diseased condition, so that it is reasonable to infer that the existence of diseased teeth was rare, from the fact of such men reviewing much concerning them without treating of such a circumstance.

We are then justified in the assertion, that the universal prevalence of diseased teeth at the present time, is owing to some cause of more modern introduction, and notwithstanding the great Hippocrates formed some inefficient compounds for "fixing" the teeth, we find but little proof of the existence of a want or necessity for their application. That this is attributable to the better understanding of what constituted natural living, is not to be entertained for a moment, but to the unsophisticated mode of life, originating in the absence of effeminating luxuries or debilitating vices, for we find immediately upon their introduction, disease following inseparably in their train, and among the most prominent, is that of the teeth.

The formidable increase of general disease and the shortness of life, with the startling decrease in man's physical happiness, is owing to the ignorance of the laws which regulate the economy during infancy, and to this, is also to be referred the decay and premature destruction of the teeth, if not entirely, to a very great extent.

That the race has greatly degenerated, is evident in nothing more forcibly than in the difficulties almost always presented in the function of child-bearing, which has become almost a disease requiring treatment in many cases before and after labor, instead of as formerly, a healthy and natural process, wanting nothing but its fulfilment to develop the nature of woman.

It is almost incredible to us, that many of the hardy races, still in enjoyment of unsophisticated modes of living, go through this process without the necessity for any assistance whatever, and without the least suspension of their usual industrial avocations, except it be for a moment of retiracy, sufficiently long to wrap the little stranger in a warm covering. Witness the account of travellers in Tartary, who state, that nothing is more common than for a woman on a journey to descend from her horse, retire a short distance from the path, give birth to her offspring, suspend it by a sort of shawl from her neck, remount and proceed onward, and all this, without the assistance from any living creature. Subsequent travelers confirm the fact among many Indian tribes.

Thus, we find nations in natural conditions, untainted by the luxuries of perverted tastes, strong and active, free from disease of body, and from such vices as are created by our more artificial state, whose progeny are not only longer lived, but who are stronger, larger and more healthy. Witness this condition of things in the mother of the present day, in comparison with the same state in the simple nature. She is, perhaps, frequently bled and physiced, blisters and plasters used, obliged to keep her bed a great part of the time, her appetite is so dainty, that nothing will satisfy her cravings, that is in the least wholesome or natural. Her duties in life cannot be fulfilled for fear of ruptures and accidents from unaccustomed positions. Nature's gambols and freaks, are all hidden from her, even nature itself shut out for fear of impressions being made upon the fetus through the great susceptibility of the mother's nerves, and her usual avocations mostly suspended, and all these miseries arise merely from a direct opposition to the laws of health.

Hereditary tendencies are strong and almost indelible, but they can be so carefully avoided and directed, as to become scarcely perceptible and wholly harmless, by such care and attention as are within the reach of every one possessed of ordinary intelligence, and a sufficient character, to follow the dictates of reason; for the dangers of all tendencies imparted at birth, are not great in themselves, but only so, as they are encouraged by silly customs of society, or by the weaknesses of parents, granting to their children the luxuries which brought these very evils into existence, or by the indulgence of habits, whose effects are opposed to the continuance or establishment of health.

By avoiding these causes, we find the aptitude to disease wholly eradicated. The physical construction of teeth may be strongly urged in opposition to these views, as to this is justly attributed the great amount of decay in the teeth. Thus, peculiar depressions, corrugations and irregularities are often transmitted through several generations, affording lodgments for the acids of the mouth, and particles of decomposing food, &c., producing the disease to which all teeth are more or less liable. But this fact is overlooked, that had the proper precautions been taken in due time, principally while the organs were in their formative stage and after eruption, the peculiarity of form would have been much changed, and their composition capable of resisting all corrosive agents, and by carefully continuing a rational system of hygiene, each successive family would fall heir to improved constitutions and more perfectly developed teeth, both as regards beauty and structure, rather than to the impaired constitutions and the irregular and defective dentures, now almost universal.

Again, it is often overlooked that the body is a unit, inseparably one, so that its integral parts cannot be effected by morbid change without implicating the whole structure. The connection may be imperceptible, but still not the less certain; for the chain of sympathy is most wisely and effectually entwined and interwoven with each tendril of the human frame, thus reciprocating each and every change of its most distant parts, so that with a defective change in the form and structure of the teeth, we have a certain indication of a defective change in some connected part of the system, which must produce an addition to the already existing causes of decay or disease in teeth which are not able to resist the before existing causes of morbid change.

Let us, for example, admit, for the sake of argument, but which, it must be remembered, is impossible, that we have a perfectly healthy constitution, but teeth defective in structure, which, with much care, will prove serviceable through life. Now, impair this constitution and the teeth will fall the first sacrifice, and by their loss will the system be less able to resist the disease by which it is oppressed, thus each change is as a compound misfortune entailing destruction upon its victim and his generations.

In the enumeration given by the most valued of dental authors of the causes of decay in teeth, it is stated to be the result of the action of chemical agents, such as vitiated saliva, putrescent particles of food, acids, corruptions of the fluid by which these organs are nourished, states of the general health, mechanical injuries, rapid variations of temperature, &c., but the worst and most fruitful source is, unquestionably, *the imperfection of structure.*

Teeth, whose constituent particles are defective in their proper relative quantities, are peculiarly subject to the action of all agents, irrespective of all other considerations, as they cannot possess the power of resistance, but yield at once to decay, so that it is an infallible evidence of the predominance of the organic material, when teeth are found affected by caries or any other disease, as it is also, of an enervated and impaired constitution; and on the contrary, teeth that are free from decay or disease at an advanced age, bear certain and undeniable proof of their possessing the proper quantity of organic and earthy salts, as well as of the existence of a sound and firm constitution.

Mahon, Delabarre, Jourdain, and others, clearly acknowledge the doctrine of great morbid sympathy existing between the teeth and the general system; that defective and irregular teeth are conclusive evidences of an unsound constitution, in our opinion, arising principally from other causes than hereditary tendencies, or from a greater one than want of cleanliness, found in the utter disregard that is universally entertained by parents, of the requisite food to be taken during the time these organs are in their embryonic stage until they are developed as perfect teeth.

It is rare, if not impossible, to find a mother who entertains the slightest opinion founded upon reason, of the food which should form her diet, before and during pregnancy, or who apprehends the least consequence to her offspring from a gloated appetite which is often so vulgarly and foolishly encouraged.

* "When a larger quantity of food is habitually consumed than the wants of the system require, it is not converted into solid flesh, but is got rid of by the various processes of excretion. The increased production of muscular fibre depends upon nothing so much as the exercise of the muscle. It cannot take place unless the blood supply it with the materials; but no degree of richness of the blood can alone produce it; consequently, the accumulation of nutritive matter in the blood is, so far from being a condition of health, *that it powerfully tends to produce disease*, either of an inflammatory character, if the fibrin predominates, or of the hemorrhagic character, if the red corpuscles predominate. This state is most apt to present itself in those who live well and take *little exercise;* and the remedy for it is either to diminish the diet, or to *increase the amount of exercise*, so as to bring the two into harmony."

To this most of our readers will doubtless subscribe, and every practitioner will be able to recall to his mind, many ludicrous tales whispered during a professional visit, of a strange fancy for this and that rarity impossible to obtain, and as impossible to digest, which is presumed to arise from the little unborn sufferer's existence, and to which is popularly yielded a consideration as hurtful as it is ignorant. It is frequently seen that where more than usual intelligence is found in the maternal guardian, a degrading concession is yielded to a depraved appetite, and a weakened system is still further taxed by poisonous indulgences, not only incapable of affording the right nutriment to the fetus, but absolutely such as will overwhelm it with corrupted matter, and that, too, at a time when the most important process is going on; for it will be remembered, that the teeth are all in such a condition as to readily receive

* Carpenter's Phy.

the least morbid impression, the permanent growing in their sac condition, and preparing the secreting vessels which deposit osseous matter during childhood, and the temporary, absolutely undergoing this operation up to the period of birth, when they are found ossified.

A very popular writer states that, "we often do our bodies great injustice by attributing to them the low appetites and perverted passions, which really belong to a degraded, spiritual nature. We see by the most striking examples, that what are called bodily indulgences, are not good for the body—are not what it demands; that simple, even coarse fare, much active exercise and exposure to what we might consider hardships, suit the body much more than luxurious habits and dainty fare."

Look at the lists of mortality amongst children; see that *one* in every *three* dies before the fifth year, and mostly those ushered into life amidst the luxuries of fashion and miserably applied wealth, certain is it then, that the condition which alone renders the child vigorous at birth, is neither understood nor sought after; a strong circumstantial proof that this mortality is the immediate result of improper living is, from its direct supervention, as hereditary entailments, even when unopposed, require time for their development.

No one, who has given this subject its needed consideration, can doubt that the physical education of the child commences with the pregnancy of the mother; and equally certain is it, that one of the greatest and most active labors of nature during this period, is in the development of the dental apparatus. The elaboration of materials for the teeth; the secretion of such an infinite admixture of foreign matters through a duplication of means, as it were; the necessity of nature's efforts to perfect, in time, this wonderful growth, and its abject dependence upon proper supply from so doubtful a source; the harmony of consent nature always requires in her perfections, which in this process, from named causes, is seldom, if ever granted; the visible struggle, reasonably inferred of the development, and of the eruption of the teeth, all go to show that nature's greatest labor in infancy is with the dental apparatus.

The remarkable and intimate relationship between the child and its parent, renders their well-being inseparable, and greatly subject to corresponding changes. The influences affecting the mother must be received by the child, and indulgences or habits which, under ordinary circumstances are, to say the least, doubtful to her, must inevitably tend to affect the child injuriously. In a word, whatever affects the health of the parent, must, as an infallible consequence, affect the child, whether the cause be of a physical or a mental nature. No longer can the fact be questioned, that improper kinds of diet, and irregularities of living in the mother, produce the most baneful effects upon the unborn child, resulting in the birth of weak and debilitated children, possessing, as a natural consequence, a defective denture. How can it be otherwise, when we find that even the mental peculiarities of the mother most strikingly affect the child. That the existence or cultivation of peculiar disposition and temper is often exhibited in the child after birth, look at the analogy of this in the lower animals.

The training of the parent is astonishingly exhibited in the young which have been entirely separated at birth, without the process of instruction, and which yet possess not only the cunning and tact, but also the voracious appetite for similar food. A writer who deserves, and will become highly popular, comments very instructively in the following manner, upon this subject: "Surely the wise mother, who can recognize the high and important use of the necessary cares, will not neglect to adopt them with a never failing accuracy. For the favoring influences affecting the offspring are constantly present in the daily life of the mother, forgetting not that a cheerful spirit, enlarging the happy prospects of the future, will throw an endless sunshine in the features of her unborn child. That these important rules of hygiene, regular habits, early hours, periodic exercise, cold bathing, plain wholesome food, loose comfortable clothing, so easily understood, so simple in their nature, so easy of adoption and so important in the favoring condition of growth, as must impress every woman of sense with their indispensable nature, and no *true mother* will allow a frivolous pleasure, petty cares, or even much cause for sorrow, to interfere with these healthy rules of life, or disturb the laws laid down in her system for the purpose that she should fill her sphere in creation."

Again, we will suppose that a child is born of perfectly healthy parents, whose care has been scrupulously directed. At birth it may have all its temporary teeth perfect in form and component parts. Its permanent rudiments are all in a perfect state. Now, if the laws regulating its food are accommodated, the condition of the one will remain in tact until nature calls for their displacement; of the other, the materials will be just and properly supplied, neither too much of the earthy or too little of the animal tissue, but that combination capable of resisting all destructive influences. The general health of the child will remain good, its whole system will be developed naturally; no febrile action from an over-taxed stomach will operate injuriously upon one organ in suspending its growth, stinting its nourishment and making it the weak point of the system; the jaws will be sufficiently developed to admit, without crowding, all the teeth in their proper position; the fluids of the mouth must be perfectly healthy, and here we have a condition of things which will allow the teeth to remain free from disease, with proper cleanliness, for twice the number of years mankind are permitted to enjoy.

That the diet of a child is closely regulative of its existence, is proved by many public facts—for instance, Mr. Thos. Bull, in his work upon the maternal management of children, states the case of a mortality so great in the work-houses of London, among the infants, as to attract the attention of Parliament, which instituted such an improvement in the matter of diet, cleanliness and clothing, as to reduce the number of deaths from 2600 yearly to 450, rendering it certain that 2150 lives were lost annually, from the traceable ignorance and mismanagement of children.

[To be Continued.]

